# Weight-Bearing Locomotion in the Developing Opossum, *Monodelphis domestica* following Spinal Transection: Remodeling of Neuronal Circuits Caudal to Lesion

**DOI:** 10.1371/journal.pone.0071181

**Published:** 2013-08-12

**Authors:** Benjamin J. Wheaton, Natassya M. Noor, Sophie C. Whish, Jessie S. Truettner, W. Dalton Dietrich, Moses Zhang, Peter J. Crack, Katarzyna M. Dziegielewska, Norman R. Saunders

**Affiliations:** 1 Department of Pharmacology and Therapeutics, The University of Melbourne, Parkville, Victoria, Australia; 2 The Miami Project to Cure Paralysis, Miller School of Medicine, University of Miami, Miami, Florida, United States of America; National Institutes of Health/NICHD, United States of America

## Abstract

Complete spinal transection in the mature nervous system is typically followed by minimal axonal repair, extensive motor paralysis and loss of sensory functions caudal to the injury. In contrast, the immature nervous system has greater capacity for repair, a phenomenon sometimes called the infant lesion effect. This study investigates spinal injuries early in development using the marsupial opossum *Monodelphis domestica* whose young are born very immature, allowing access to developmental stages only accessible *in utero* in eutherian mammals. Spinal cords of *Monodelphis* pups were completely transected in the lower thoracic region, T10, on postnatal-day (P)7 or P28 and the animals grew to adulthood. In P7-injured animals regrown supraspinal and propriospinal axons through the injury site were demonstrated using retrograde axonal labelling. These animals recovered near-normal coordinated overground locomotion, but with altered gait characteristics including foot placement phase lags. In P28-injured animals no axonal regrowth through the injury site could be demonstrated yet they were able to perform weight-supporting hindlimb stepping overground and on the treadmill. When placed in an environment of reduced sensory feedback (swimming) P7-injured animals swam using their hindlimbs, suggesting that the axons that grew across the lesion made functional connections; P28-injured animals swam using their forelimbs only, suggesting that their overground hindlimb movements were reflex-dependent and thus likely to be generated locally in the lumbar spinal cord. Modifications to propriospinal circuitry in P7- and P28-injured opossums were demonstrated by changes in the number of fluorescently labelled neurons detected in the lumbar cord following tracer studies and changes in the balance of excitatory, inhibitory and neuromodulatory neurotransmitter receptors’ gene expression shown by qRT-PCR. These results are discussed in the context of studies indicating that although following injury the isolated segment of the spinal cord retains some capability of rhythmic movement the mechanisms involved in weight-bearing locomotion are distinct.

## Introduction

Following injury the adult mammalian spinal cord shows little capacity for functional repair involving regeneration of axotomised neurites. In spite of many years of intensive research to promote such regenerative growth at the site of injury, no new therapies for patients have resulted and attempts to replicate apparently promising studies have so far failed [1]. In recent years it has become apparent that a more effective approach may be to build on much older studies of Sherrington [2] and others [3] indicating that even when isolated from the brain, the adult spinal cord retains the ability to produce rhythmic activity of the limbs when they are stimulated by sensory input. However, such rhythmic activity is generally not accompanied by an ability to support body weight, except in some animal models after intensive training (e.g., spinalised cats, [4], [5]; see [6] for review of different species).

Extensive studies have given rise to the notion of a central pattern generator (CPG) in the lumbar spinal cord with the intrinsic ability to generate rhythmic movements of the hindlimbs 7–9. Further studies have suggested that in spinal animals, these CPGs may be able to be activated when there is peripheral sensory input via the hindlimbs [10]. Systematic attempts to use this information in patients consisted of clinical trials in spinal cord injured individuals whose body weight was supported in a harness over a treadmill. The results, however, did not show any consistent improvement in unsupported body weight locomotion compared to overground walking therapy [11]. In both animal and patient studies body weight supported treadmill training has been extended by use of a feedback robot, but in patients a recent systematic review concluded that the evidence for efficacy was lacking because of inadequate experimental design [12], [13]. A few robotic systems have also been developed for animal studies [14], [15]. However, it seems that the mechanisms involved in rhythmic limb movements originating from central pattern generators and the mechanisms involved in body weight support are distinct. Thus loss of supraspinal input in spinal cord injury results in loss of body weight support that, except in particular specific circumstances [6], does not recover following treadmill training with or without robotic assistance. The addition of electrical stimulation of the spinal cord together with administration of drugs (e.g., agonists for serotonin, dopamine and noradrenalin receptors) appears to be required to raise the state of excitability of the propriospinal neural circuits that are responsible for weight bearing in a normal individual (see [6], [10], [16]).

These approaches are quite complex and time consuming for the patients and it has yet to be shown how applicable they will be in the wide range of injuries sustained following trauma to the spinal cord. As an alternative approach we have followed up on our recent report on the ability of the Grey short-tailed opossum (*Monodelphis domestica*) to exhibit weight bearing locomotion when injured in the postnatal period, an ability that is possible even when there is no evidence of axonal growth across the site of a complete spinal transection made at 4 weeks of age and without any interventions like treadmill training, drugs or electrical stimulation [17]. Lesions made earlier (one week of age) were followed by developmental and regenerative axon growth across the lesion with subsequent development of essentially normal locomotor function [17], [18]. This has also been demonstrated *in vitro* in preparations of cultured *Monodelphis* spinal cord, where axon growth across a spinal lesion occurred following injury in the neonatal but not in two-week-old spinal cords [19]–[21]. Similar findings of axon growth across a lesion, providing it had been made early in development, have been reported in North American opossums, *Didelphis virginiana* [22]. Marsupial species such as *Monodelphis* and *Didelphis* are particularly favourable for studies of the response of the developing spinal cord to injury because the animals are born at a very early stage of development [23]. A response involving axon growth across a spinal cord injury in eutherian mammals such as rodents has only been described in fetuses [24]–[26]. Rodents with spinal cord injuries made in the first week of life do show some limited locomotion with only variable body weight support [27]–[29], but the recovery is also not associated with axon growth across the lesion [30].

In the present study we have focused on the development of weight bearing locomotion in *Monodelphis* transected at two different stages of spinal cord maturity: at an age (4 weeks) when no axon growth occurs across the lesion (more akin to the clinical situation in humans with severe spinal cord injuries) and compared these spinal animals with those transected at an even earlier stage of development (one week) when supraspinal innervation is restored [17]. For reasons of experimental consistency we have used a transection injury rather than a contusion, which would have been more like the clinical situation The ability to walk with weight bearing steps in spite of a lack of axonal connections across a spinal cord lesion is an interesting fundamental property of the spinal cord at a particular stage of its development. Understanding the biological basis for this ability might eventually lead to development of less complex interventions in spinal cord injured patients than are currently being contemplated.

## Methods

### Ethics Statement

The University of Melbourne Animal Ethics Committee approved all animal experiments described in this study, under ethics no. 111198. All experiments were conducted following National Health & Medical Research Council (NH&MRC) guidelines.

### Animal Care

South American Grey short-tailed opossums, *Monodelphis domestica,* were supplied from a breeding colony maintained by the Biological Research Facility (BRF) at the University of Melbourne. A comprehensive description of these animals’ husbandry has been published previously [23], [31], [32] and is only outlined briefly here. Opossums are housed in polycarbonate boxes in temperature- and humidity-controlled (27°C; 60% humidity) rooms in the Animal House facility with a 14∶10 hour inverted light/dark cycle. Food and water are given *ad libitum.* Following a gestational period of 13 days mothers give birth to litters of 5–8 pups, which remain attached to the maternal teats until approximately post-natal day (P) 14, after which they start to detach for increasing time intervals. Pups are weaned at P60–65 and begin to reach sexual maturity at about 5 months of age.

### Numbers of Animals used in this Study

In total 27 animals were used in this study. Control (n = 11), P7-injured (n = 7) and P28-injured opossums (n = 9) were all allowed to grow to adulthood (P90–110) before being assessed using a series of behavioural tests (see below). Following these tests, animals were either used for retrograde labelling or gene expression analysis as described below. Table 1 summarises numbers of animals used for the different experiments.

**Table 1:** Summary of animals used in this study.

Group	Behaviouraltesting	Retrogradetracing	Gene expression
Control	11	5*	4
P7-injured	7	3*	3
P28-injured	9	4^#^	4

Behavioural testing was performed in all animals in each group. Following this animals were divided between two groups: retrograde tracing or PCR. *Note: animals whose tracer injection site was deemed unsatisfactory (see methods, below) were not included in tracing analysis. ^#^Note: one P28-injured opossum underwent spinal re-transection in adulthood and consequently was not included in retrograde tracing counts.

### Anaesthesia and Post-operative Care

Surgical procedures were similar to those described previously [17]. Anaesthesia was induced using inhaled isofluorane. Animals were anaesthetised to a surgical level and maintained with 3–4% isofluorane in O_2_-enriched air, and all procedures were performed under sterile conditions. For the duration of the operation animals were placed on a heated pad (25–28°C). During recovery from anaesthesia opossums were placed under a heat-lamp. Post-operative pain was managed using intraperitoneal injections of buprenorphine (0.06 mg/kg). No post-operative infections were observed. No manual voiding of the bladder was required for any animal in this study.

### Spinal Cord Injuries

Spinal cord injuries were made at two different ages, post-natal day (P) 7 or P28. Although detailed procedural methodology has been described previously [17], a brief description follows here.

At P7 pups are still attached permanently to the mother’s teats making it necessary to anaesthetise the mother for the duration of the surgery. After anaesthetic induction the mother was placed supine to expose the pouchless abdominal area to which the pups attach. In addition, P7 pups were individually anaesthetised by placing over their snout a 1.5 mL tube filled with isofluorane-soaked cotton wool. The skin overlaying the mid-thoracic vertebrae was cut and using a fine ophthalmic blade (Sharpoint, 15° stab blade) a laminectomy was performed to expose the spinal cord. Spinal transection was made using fine scissors (5 mm blade, Fine Science Tools). Completeness of the transection was confirmed by running the point of a fine ophthalmic blade through the lesion site in contact with the spinal bones to ensure that no tissue bridges remained. Following tissue closure, the wound was sealed with surgical glue (Vetbond tissue adhesive, 3 M) and washed thoroughly with sterile saline. After recovery from anaesthesia the mother was returned to the animal facility. Pups injured at P7 heal very quickly and no tissue scar forms making identification of injured pups difficult, therefore all pups in a litter were subjected to spinal injury, while age-matched un-operated animals from different litters served as controls.

By P28 pups detach from the mother’s teats for increasing periods of time and can be removed from the cage for surgical interventions. Pups were individually anaesthetised using inhaled isofluorane delivered via a facemask and were maintained on a heated pad under continuous anaesthesia. A longitudinal incision was made in the skin overlying the lower thoracic vertebrae, the musculature of the spinal column was incised to reveal the spinal column and a laminectomy was performed at T10. The spinal column was stabilized throughout using a stereotaxic frame. The spinal cord was completely transected using a fine ophthalmic blade, which was then passed repeatedly through the injury site to ensure no tissue bridges remained intact. Once the transection was done, the skin incision was sealed using surgical glue. Control animals (from the same litter) were anaesthetised but no operation was performed. All pups were returned to their mother after they had recovered under a heat lamp for 1 hour.

We have confirmed previously the effectiveness of this lesioning technique (always performed by the same operator; BJW) by randomly selecting P7 and P28 pups immediately after surgery and fixing the spinal cords for histological analysis which consistently showed that spinal transections were complete (see [17]).

### Behavioural Studies

All animals grew to adulthood in their normal environment and were not interfered with further until they had reached 90–100 days of age when their locomotor abilities were assessed using an array of behavioural tests that we have used and described previously [17], [18].

#### Open field locomotion

The Basso, Beattie, Bresnahan (BBB) Locomotor Rating scale [33] was used to assess animals as they moved about freely in a standard polycarbonate animal box (30 cm×40 cm) with a smooth non-slip floor. The Locomotor Rating scale was developed for use in rat models of spinal cord contusion and transection but has been adapted for use in the two species of opossum, and published previously [17], [22]. *Monodelphis domestica* are a furtive species; consequently, it was found that attempts to assess them in large open spaces (as recommended in the original BBB publications) led to the animals running too rapidly from point to point for fine assessments to be made of limb placement and coordination. Therefore the size of the assessment area was modified (30 cm×40 cm) and this resulted in more accurate assessment due to more controlled, slower movements of the animals. Two blinded assessors observed *Monodelphis* for 4 minutes as they moved about the box and a score on the 21-point BBB scale was assigned. The test was performed under low ambient light and the animals were encouraged into constant exploratory locomotion by gently tapping the sides of the box.

#### Gait Analysis

Digital video recordings were made as *Monodelphis* walked on a treadmill moving at 6 metres per minute. A single camera was placed at 90° to the direction of locomotion (i.e., perpendicular to the sagittal plane of the animal). No modifications in treadmill speed were necessary for any injured animal. Footage (taken at 30 frames/second) was manually analysed frame-by-frame and each paw placement and lift-off was plotted from a continuous period of 8–10 seconds and a representative analysis period (typically encompassing 6–10 step cycles) was selected that was free of pauses in gait and other anomalies. Gait parameters were computed from these gait traces. Regularity Index (RI) was calculated by expressing the number of normal step sequence patterns (NSSPs; the sequential use of all four limbs in any order) as a percentage of total number of steps taken. Total step cycle, stance and swing durations were also calculated together with phase lags. Phase lag is defined as the point in time during the total step duration of one limb that another limb is placed. Phase lags can be calculated for any pair of limbs, but here, since opossums mostly use an alternating gait pattern (–LF–RH–RF–LH–; [34]), only “in sequence” and girdle phase lags were calculated.

#### Swimming test

Swimming tests an animal’s ability to move its limbs in an environment of reduced sensory feedback [18], [35], [36]. *Monodelphis* were recorded as they swam lengths of a narrow swimming pool (100 cm long×20 cm wide×60 cm deep) and climbed onto a visible platform placed at one end. The video footage was later examined for any indication of hindlimb movement during swimming, which would indicate the involvement of supraspinal control [17], [18].

### Retrograde Axon Tracing Studies

Following behavioural testing, quantitative anatomical tracing was employed to examine to what extent neuronal pathways had been remodelled following spinal injury in the neonatal period.

#### Tracer injection

A unilateral injection of fluororuby (tetramethylrhodamine-labelled dextran amine, 10,000 MW; Molecular Probes) was made into the lumbar spinal cord (L1/2) below the injury site, as described previously [17], [37]. Briefly, in deeply anaesthetised animals, after blunt dissection of the musculature overlying the spinal column, a small hole was drilled through the dorsal plate on the right side of the L1/2 vertebra through which a single injection of fluororuby (0.55 µl per injection; 25% w/v dissolved in 0.1 M Tris buffer with 2.5% (v/v) Triton X-100) was made using a pulled glass micropipette (70 µm outer diameter) under gentle pressure. The area was washed with sterile saline and packed with Gelfoam and the wound was sealed using number 4.0 silk sutures (Ethicon) and tissue glue. Animals were killed 6 days later and perfuse-fixed with ice-cold paraformaldehyde (4% in PBS). Brains and spinal cords were dissected and post-fixed in the same fixative overnight at 4°C.

#### Neuronal counting and mapping

Following post-fixation the brains and spinal cords were embedded separately in 4% agar blocks. The spinal cord was divided into smaller segments as appropriate for embedding and analysis purposes (see Results below). Brain and spinal cord tissue was sectioned at 100 µm using a vibrating microtome (Leica VT1000S) and mounted on glass slides under Fluorescent Mounting Media (DAKO). Brain was sectioned in the coronal plane and spinal cord was sectioned in the transverse plane. Sections were stored at 4°C. All labelled cells were counted manually in sections viewed under an appropriate filter specific to the wavelength of the fluorophore (Texas Red filter, Olympus BX50 microscope with a DP70 digital camera attached). For brainstems, all sections were analysed and every fluorescently labelled neuron was morphologically identified and manually counted under 20×magnification [17], [37], [38]. For spinal cords, 10 evenly spaced sections through each area of interest were manually counted (as above for brainstems) and these counts were pooled. Additionally, fluorescent neurons in all analysed spinal cord sections were mapped using the layer function in Adobe Photoshop, where each dot represented one labelled neuron. These neuron maps were then overlaid to produce 2-dimensional reconstructions of fluororuby-labelled neurons throughout the areas of interest. These overlays were not used for counting purposes, but were instead used to give a general map of the distribution of labelled neurons.

#### Morphometric analyses

After fluorescent neurons had been counted, three sections per spinal segment were used for morphometric measurements and analysed under light microscope where grey and white matter areas can be clearly distinguished. Total section area and separate white and grey matter areas of each cross-section were determined. These values were then plotted to give a geometric profile of cross sectional areas along the entire spinal cord.

### Gene Expression Studies: qRT-PCR

Following behavioural assessment, a subset of animals (see Table 1) was killed and the spinal cord was dissected out, divided into segments (using the same divisions used for propriospinal neuron counts) and snap frozen in liquid nitrogen. Gene expression studies were performed on two of these samples: the L1–2 and L3–5 segments, both caudal to the injury site (which was performed at the T10 level; see Methods).

Total RNA was extracted using the Trizol method [39] and quantified using a NanoDrop ND-1000 UV-VIS spectrophotometer (Thermo Scientific). Quantitative real-time PCR (qRT-PCR) was used to determine the expression of selected neurotransmitter receptor genes. Reverse transcription from RNA to cDNA was performed where a 9 µl aliquot was used for samples with the lowest concentration (up to 2 µg total RNA) and other higher concentration samples were diluted to match to the lowest sample. qRT-PCR was performed on all samples using RT^2^ SYBR Green ROX qPCR Mastermix (Qiagen) using gene specific primers based on published sequences for both *Monodelphis domestica* and human genome. Three genes from each class of receptors: excitatory, inhibitory and modulatory were chosen. Table 2 shows forward and reverse primers used for the genes analysed in this study. The design of each primer was checked for efficiency by running standard curves and dissociation curves on all plates. The reaction was performed using an Applied Biosystems 7900 HT Real Time System. All primer pairs were tested for specificity (by dissociation curves). Cyclophilin was used as a general internal control reference gene for whole tissue. Average threshold cycle (Ct) values were obtained from triplicates for each sample. Analysis was completed for each sample by normalizing this average Ct value for each gene to its internal cyclophilin Ct value (to give the corresponding ΔCt value) followed by a comparison between spinal cord injured and control animals (to give ΔΔCt values). Fold changes were calculated relative to control using the 2^−ΔΔCt^ calculation and expression ratios (Log_2_ transformations) used to express these changes graphically.

**Table 2:** Primers used in this study.

Name	Gene	Forward primer sequence	Reverse primer sequence
**Cyclophilin**	*Ppia*	TCCAAAGGCAGCAGAAAACT	AAAACTGGGAGCCATTTGTG
**NMDA-1**	*Grin1*	ACCCGCATGTCCATCTACTC	TCCAGCTGTAGACACGCATC
**AMPA-2**	*Gria2*	TCGAGCAAAGAAAACCCTGT	GCTGACCTTGGAATCACCTC
**mGlu-R5**	*Grm5*	AAATCCTCCAGTCGGGAACT	TTTTGGTGACCAGTGCAGAG
**GABA_A_-R β2 subunit**	*Gabrb2*	GACAACAGAGTGGCAGACCA	ATAAAGGACGGTGCCATCAG
**Glycine-R α1 subunit**	*Glra1*	TGTCTCCCTCGGATTTTCTG	AGGGTCATTCCACTGCTGAC
**Glycine-R β subunit**	*Glrb*	GGAAGGGGAAAAAGAAGCAG	ATTCCATTTCTGCCTCAGGA
**5-HT_1a_ receptor**	*Htr1a*	TATGATGCCCAACGTCTTGA	GCAACTCCAAGGACCACCTA
**5-HT_2a_ receptor**	*Htr2a*	ATATGCTGCTGGGTTTCCTG	TGGGGTTCCGAATAGCAATA
**5-HT_7_ receptor**	*Htr7*	CGATCATGACCCTCTGTGTG	TAGCCAAAGTCCTGGCTGAT

Designed with reference to *Monodelphis domestica* database **(Ensembl database MonDom5** http://www.ensembl.org/Monodelphis_domestica **)**.

### Statistical Analyses

Data are presented as mean ± sem. All data were plotted and analysed using GraphPad Prism software. For the majority of studies using multiple comparisons One-way Analysis of Variance (ANOVA) was used for analysis, with a Tukey post-test where appropriate.

## Results

In order to establish to what extent functional recovery had occurred following a complete spinal transection at either P7 or P28 the behavioural characteristics of spinally injured *Monodelphis* were determined and compared with age-matched control animals. Following the completion of behavioural testing, propriospinal connections in the spinal segment caudal to the original injury were investigated using fluorescent axonal tracing probe, fluororuby. Finally gene expression studies were performed on segments caudal to the injury site in an attempt to identify the pattern of changes in neuronal innervation that could explain locomotor abilities of spinal *Monodelphis*.

### Behavioural Testing

**Figure 1:**
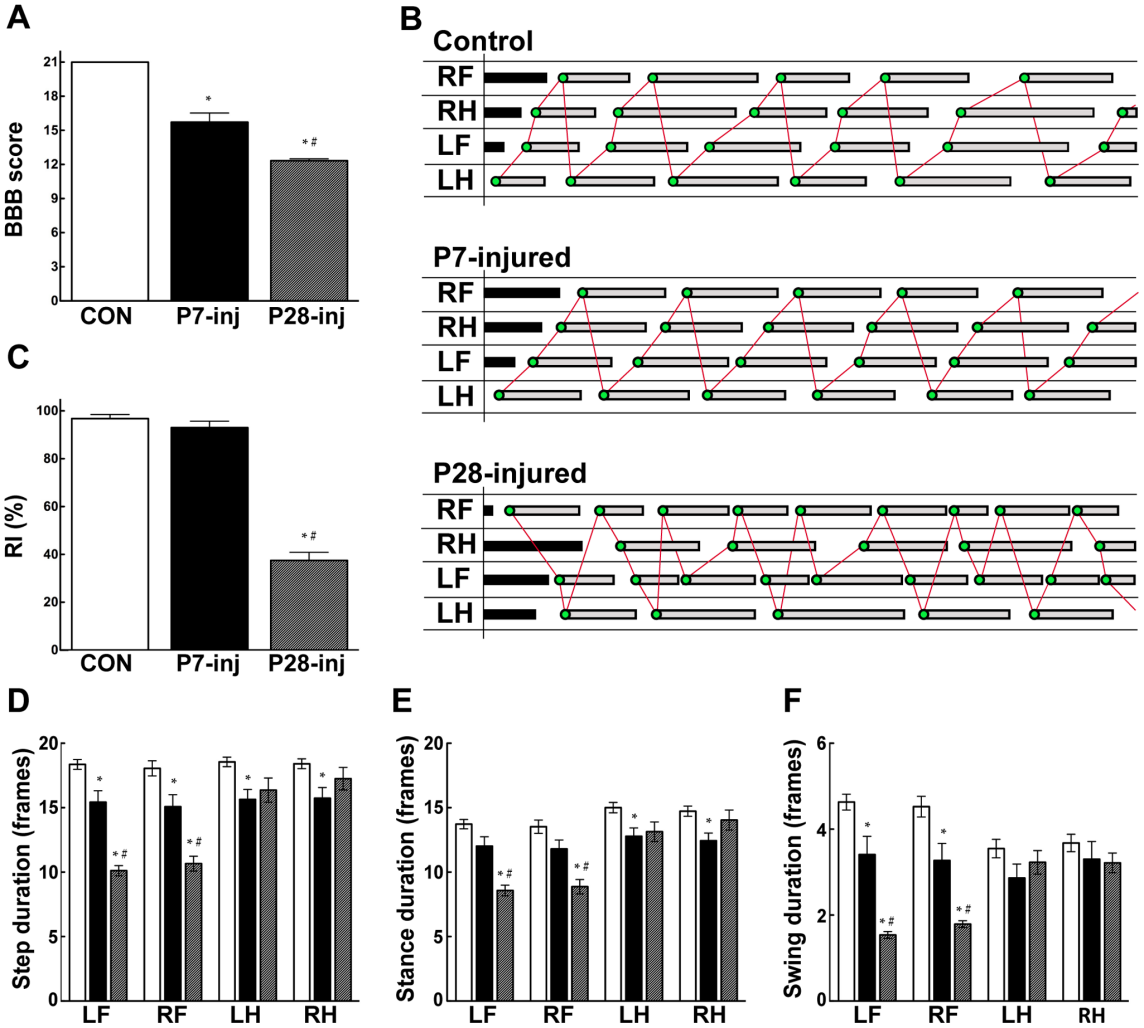
Behavioural assessments. Control (n = 11, white bars), P7-injured (n = 7, black bars) and P28-injured (n = 9, grey bars) opossums. ***A***: BBB Locomotor rating scores. ***B***: Representative gait traces from treadmill locomotion. ***C***: Regularity index. *D*: Step duration. *E*: Stance duration. *F*: Swing duration. Mean ± sem; *P≤0.05 vs control; ^#^ *P*≤0.05 vs P7-inj, by One-way ANOVA.

Open field locomotion was assessed using the semi-quantitative BBB scale [17], [33] and the results are illustrated in Fig. 1A. All opossums injured at either P7 or P28 were able to take body-weight supporting steps with their hindlimbs. All P7-injured animals achieved consistent FL–HL coordination, although trunk instability and rotational errors in placing the hind paws led to a BBB score of 15.7±0.8. This was significantly different from the uninjured control group, which showed normal locomotion (BBB = 21). In contrast to P7 injured animals, no *Monodelphis* injured at P28 achieved consistent FL–HL coordination in spite of the full weight supported plantar stepping. These animals scored 12.3±0.2 on the BBB scale, significantly different from both control and P7-injured opossums (Fig. 1A).

To further assess the opossums’ locomotion (Fig. 1B–F), video footage of these animals walking on a treadmill was analysed frame-by-frame to produce gait traces (Fig. 1B) from which coordination and other gait characteristics could be computed. Regularity index (Fig. 1C) measurements showed that both control (96.8±1.7%) and P7-injured (93.0±2.7%) opossums walked with highly regular FL–HL paw placement coordination, but P28-injured opossums did not (37.5±3.4%). In the P28-injured opossums this decrease in coordination reflected the fact that animals took different numbers of FL and HL steps leading to a FL:HL step ratio of 1.7∶1; this was in contrast to control (1.01∶1) and P7-injured (1.02∶1) opossums, where each forelimb and hindlimb was used equally. This inequality of limb usage following injury at P28 resulted from a shortening of step duration in the forelimbs relative to controls, rather than any change in the durations of the hindlimb steps, which did not differ from controls (Fig. 1D). In P28 injured animals stance and swing phases of forelimb stepping were both altered but no change was observed in either stance or swing duration in the hindlimbs (Fig. 1E–F). P7-injured animals displayed step durations that were slightly decreased for all limbs compared with controls (Fig. 1D–F). This resulted from a decrease in swing duration for the forelimbs, but for the hindlimbs the decrease was in stance duration (Fig. 1E–F).

**Figure 2:**
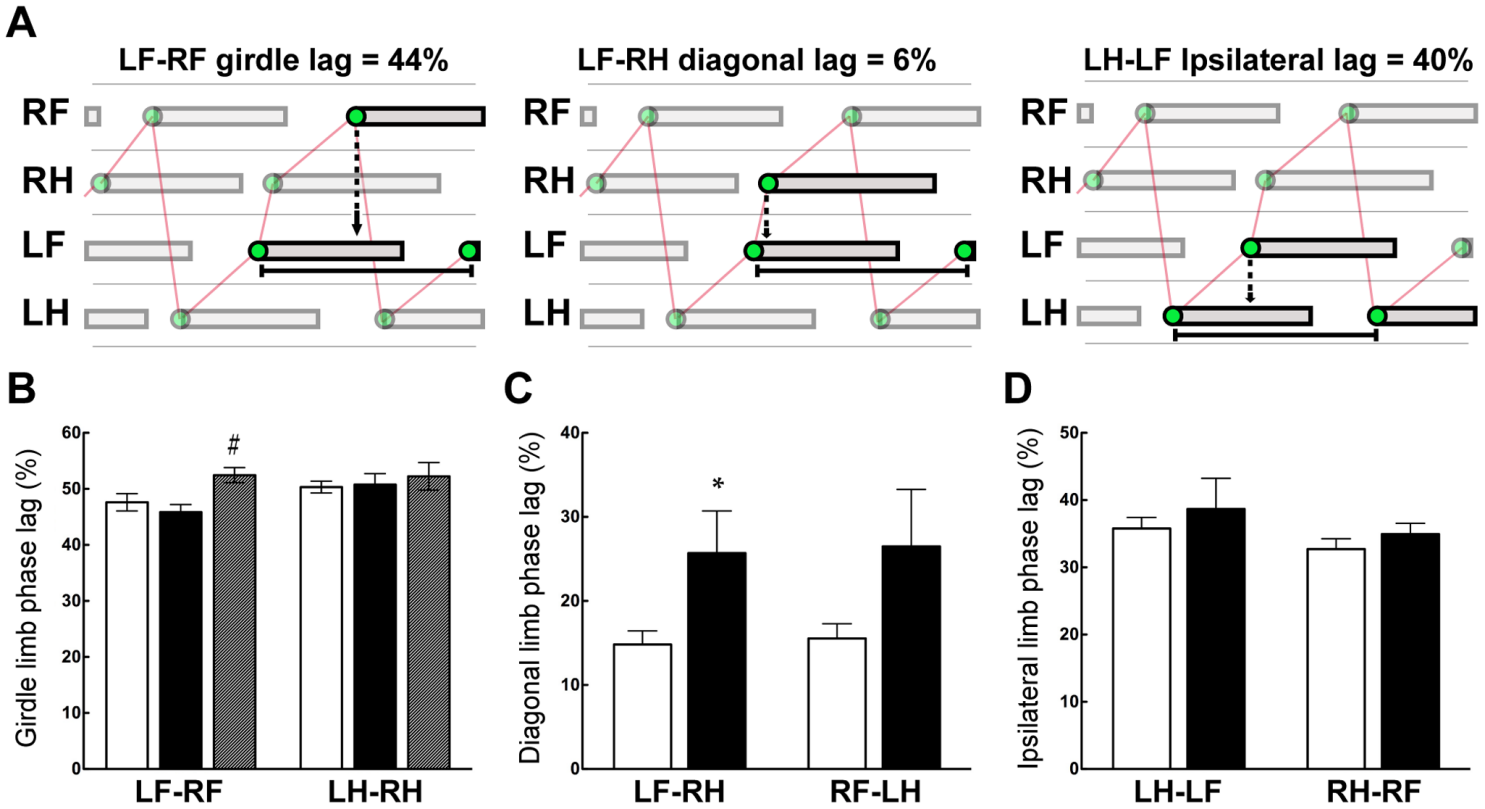
Limb placement phase lags. Control (n = 11, white bars), P7-injured (n = 7, black bars) and P28-injured (n = 9,grey bars) opossums. ***A***: Schematic examples showing calculation method for girdle, diagonal and ipsilateral phase lags. These examples are taken from the control animal in Fig. 1B. ***B***: Forelimb and hindlimb girdle phase lags ***C***: Diagonal phase lags. ***D***: Ipsilateral phase lags. Mean ± sem; *P≤0.05 vs control; ^#^ *P*≤0.05 vs P7-inj, by One-way ANOVA.

The Regularity Index assesses the order in which paws are placed but does not take into account the timing of their placements. For this purpose phase lags were determined (Fig. 2A). Results are shown in Fig. 2B–D and summarized briefly here. No differences were found for either fore or hindlimb girdle phase lags (Fig. 2B) for either injury group when compared to controls. However, the forelimb girdle phase lag was increased slightly in P28-injured animals compared with P7-injured animals. Since P28-injured animals recorded very few normal step sequence patterns (NSSPs) and, when they did, seldom employed any one repeated gait pattern, no other phase lags (diagonal or ipsilateral) were calculated for this group of animals. The control and P7-injured animals on the other hand, were highly regular in their use of an alternating gait (AltB; [34]), so “in-sequence” phase lags were calculated for these groups of animals. The diagonal lags LF–RH and RF–LH both appeared to be increased following injury at P7 (Fig. 2C), though only LF–RH was statistically significant. No differences were found for ipsilateral lags between control and P7-injured opossums (Fig. 2D).

Examination of footage of swimming opossums allows their assessment in an environment of low sensory feedback, reducing the influence of peripheral reflexes on hindlimb movements [17], [18]. This is a critical measure of an animal’s capacity to make supraspinally mediated movements of the hindlimbs. Opossums are strong swimmers and typically use all four limbs during swimming [18]. P7-injured opossums also used all four limbs and were likewise able to swim well. P28-injured opossums, in contrast, did not use their hindlimbs when swimming and relied only on their forelimbs to propel them forwards. Once these P28-injured animals touched the submerged portion of the exit platform with their hindlimbs, they were able to climb out of the pool and use their hindlimbs for weight-bearing locomotion.

Results from these locomotor and swimming studies confirmed similar observations from a different previously published study, where representative video footage can be seen [17].

### Brainstem Neuron Labelling

In order to determine if any axons had grown across the lesion site or if any spinal cord circuits had remodelled following injury, quantitative retrograde neuronal labelling was conducted in a subgroup of each of the three groups of animals studied (control, P7-injured and P28-injured; see Table 1 in Methods) at completion of behavioural testing (above). Fluororuby was injected into the right hand side of the spinal cord at L1–2 and the spinal cord and brainstem were examined for fluorescently labelled cell bodies (see Methods). Brainstems were examined to establish whether any supraspinal fibres had crossed the injury site (see Fig. S1). The brainstems of control and P7-injured animals showed many labelled cell bodies (total numbers not significantly different between these two groups) suggesting pronounced regrowth of brainstem fibres across the injury site after spinal transection at P7. In contrast, no fluorescent labelling was detected in the brainstems of P28-injured animals suggesting no regrowth across the lesion was present following injury at this age. These data closely match previous, more extensive observations of brainstem labelling using bilateral fluororuby injections [17].

### Morphometric Analysis

**Figure 3:**
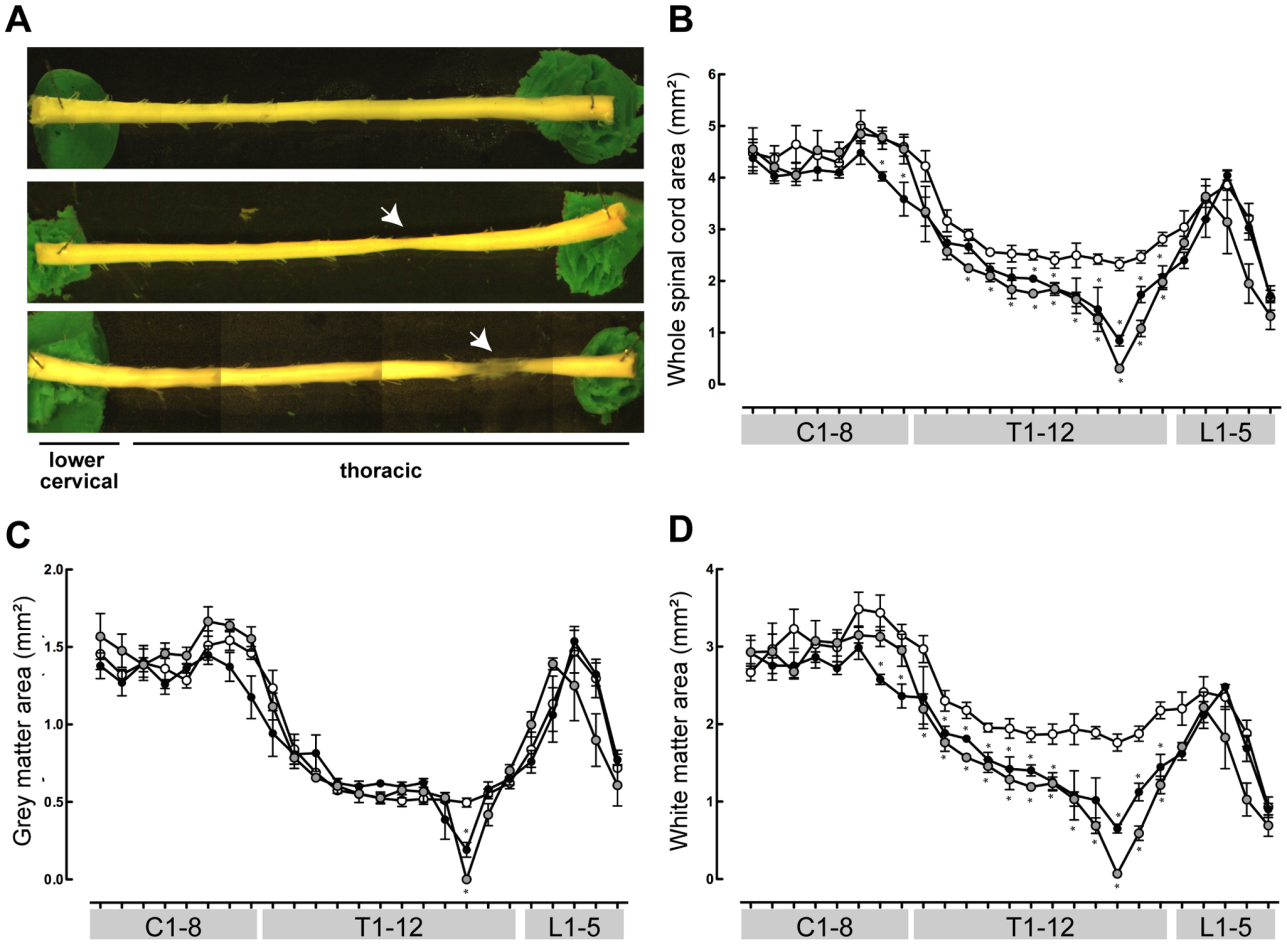
Morphometric measurements of *Monodelphis* spinal cords. Control (n = 5, white circles), P7-injured (n = 3, black circles) and P28-injured (n = 4, grey circles) opossum spinal cords. ***A***: Representative whole mounts of lower cervical and thoracic spinal cords from control (top), P7-injured (middle) and P28-injured (bottom) *Monodelphis*. ***B***: Whole spinal cord cross-sectional area along the length of the spinal cord. ***C***: Grey matter area. ***D***: White matter area. Mean ± sem. *P≤0.05 vs control. Stars denoting significance appear immediately *above* data for P7-injured animals or immediately *below* data for P28-injured animals.

**Figure 4:**
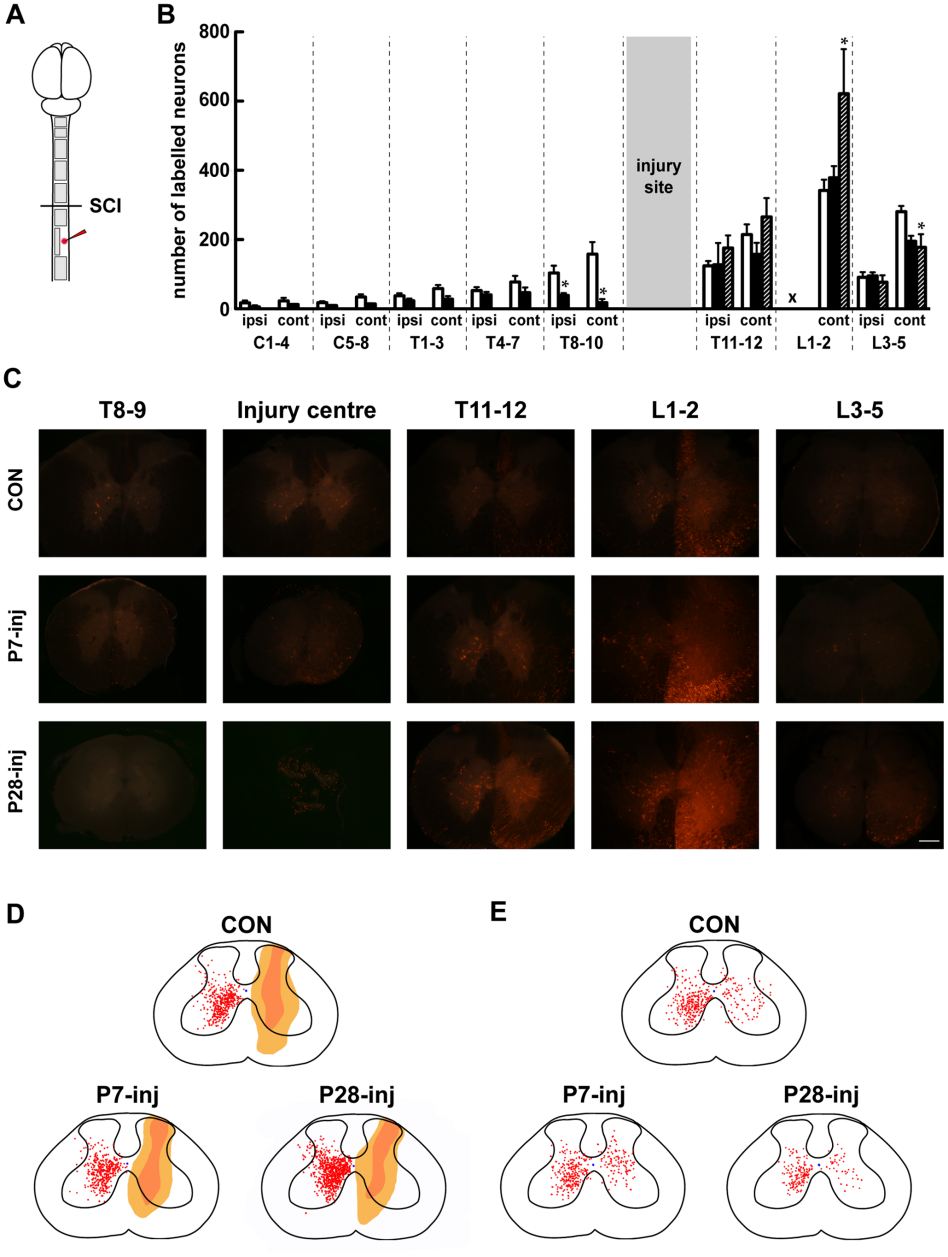
Propriospinal labelling in the spinal cord. ***A*** *:* Schematic of labelling protocol and areas of interest. ***B***: Labelled neurons in spinal cord of control (white bars), P7-injured (black bars) and P28-injured (grey bars). ***C*** *:* Representative images of labelled neurons in the lower spinal cord. Note the lack of labelling rostral to the lesion in the P28-injured cord, indicating that no axons from propriospinal neurons crossed the lesion site. ***D*** *:* 2D reconstruction of labelling in the L1–2 spinal segments. This was the level at which marker was injected. The extent of they dye in the ispilateral cord is indicated; no neuron counts could be made in this area because of the presence of injected fluorescent marker. ***E*** *:* 2D reconstruction of labelling in the L3–5 spinal segments. Mean ± sem; *P≤0.05 vs Control, by One-way ANOVA.

Morphological measurements were made along the entire length of the spinal cords dissected out from animals at the completion of behavioural and axon tracing studies (Fig. 3). Whole mounts of control, P7- and P28-injured spinal cords are shown in Fig. 3A. It was apparent that P7-injured spinal cords had regrown dense tissue across the injury site, but in P28-injured animals loose, transparent tissue had bridged the site of transection. Serial sections that were cut for labelled propriospinal neuron counting (see Fig. 4) were examined and morphometric measurements were computed for all spinal segments. Under light microscope, a clear distinction between grey and white matter is visible in these sections without further staining; results are shown in Fig. 3B–D. Whole cord cross-sectional area was decreased at the injury site of both P7- and P28-injured opossums (Fig. 3B) compared to controls. In both injured groups this decrease in cross-sectional area appeared to persist throughout the majority of the thoracic spinal cord but in the lumbar and cervical regions the cross-sectional area between the groups was no longer different. There was a sharp, significant decrease in grey matter area but this only occurred at the site of injury in both injured groups and quickly returned to normal levels where it remained closely matched to the control cords for the much of the remainder of the spinal tissue (Fig. 3C). The pattern of white matter area changes showed a persistent decrease throughout the thoracic cord that was even more pronounced than that of the whole cord cross-sectional area. The values approached those of controls in both cervical and lumbar segments (Fig. 3D).

### Propriospinal Labelling

In order to identify regrowth of, or modifications to, neuronal pathways that could be involved in the locomotor recovery of spinally injured animals, fluororuby was injected unilaterally into L1–2 spinal cord (see Methods) and labelled neurons were counted in sections throughout the spinal cord (Fig. 4A). Results of these counts are shown in Fig. 4B and representative images are shown in Fig. 4C. In control animals fluorescently labelled neurons were found in every spinal segment of the cord (Fig. 4B–C). These were located both ipsilateral and contralateral to the injection site. Rostral to the injury site, labelled propriospinal neurons were at all spinal levels in P7-injured animals, though fewer in number than in controls. No fluorescently labelled neurons were found in any spinal segment rostral to the injury site in the P28-injured animals (Fig. 4B–C). However, caudal to the injury site in the contralateral grey matter of L1–2 segments an increase in the number of labelled neurons in P28-injured but not P7-injured animals, compared to controls was found (Fig. 4C). In the L3–5 segments, on the other hand, a decrease in the number of labelled neurons in the contralateral grey matter for both P7- and P28-injured animals, compared to controls was observed. No obvious differences in the distribution of the labelled neurons were found in either the L1–2 (Fig. 4D) or L3–5 segments (Fig. 4E).

### Gene Expression: qRT-PCR

**Figure 5:**
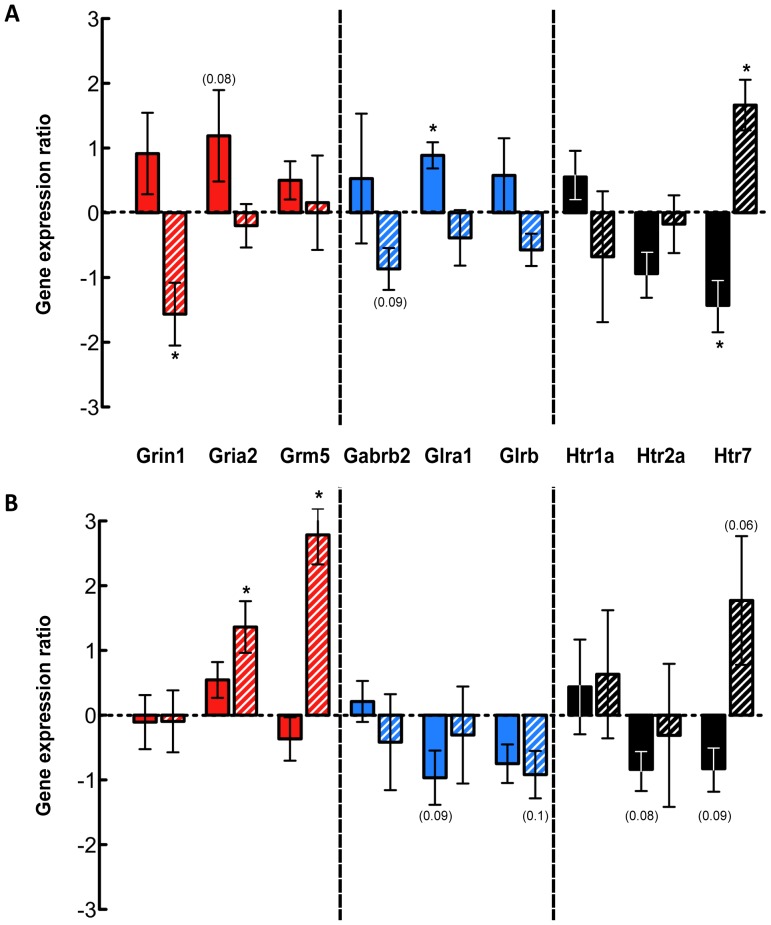
Gene expression quantitation by qRT-PCR for L1–2 and L3–5 spinal segments. Gene expression changes in the spinal cords of P7- (solid bars) and P28-injured (hatched bars) opossums are shown relative to control expression. ***A***: Gene expression ratios for the L1–2 spinal segments. ***B***: Gene expression ratios for the L3–5 spinal segments. All data are mean ± sem. *P≤0.05; *P* values ≤0.1 are indicated in parentheses.

In order to identify the characteristics of propriospinal neuron cell populations in the lumbar spinal cords of control and injured opossums qRT-PCR was employed to examine gene expression of selected neurotransmitter receptors in the lumbar cord, caudal to the injury site. Gene targets were chosen to incorporate 3 receptors each involved in excitatory, inhibitory and neuromodulatory signalling, and included receptors of glutamate, GABA, glycine and serotonin. Results are shown in Fig. 5.

#### Excitatory neurotransmitters

Quantitation of the expression of three excitatory neurotransmitter receptor genes *Grin1* (NMDA receptor 1), *Gria2* (AMPA receptor 2) and *Grm5* (metabotropic glutamate receptor 5) is shown in Fig. 5A & B (red bars). In the L1–2 segments (Fig. 5A), all three excitatory neurotransmitter receptor genes in P7-injured opossums were moderately upregulated compared to uninjured controls, though none reached statistical significance. For P28-injured animals the expression of *Grin1* was decreased significantly, while *Gria2* and *Grm5* did not change. A different pattern of expression was observed in the L3–5 cord segment (Fig. 5B). *Gria2* and *Grm5* expression was significantly increased in P28-injured animals; but neither changed in P7-injured opossums. *Grin1* expression was not different from controls in either P7 or P28 injury groups.

#### Inhibitory neurotransmitters

Quantitation of the expression of the three inhibitory neurotransmitter receptor genes, *Gabrb2* (GABA-A receptor beta-2 subunit), *Glra1* (Glycine receptor alpha-1 subunit) and *Glrb* (Glycine receptor beta subunit) is shown in Fig. 5A & B (blue bars). In the L1–2 segments (Fig. 5A), the expression of all three inhibitory receptors in P7-injured animals appeared higher than controls although only expression of *Glra1* was statistically significant. For P28-injured opossum in this segment all appeared to be downregulated, though none reached statistical significance. In the L3-5 segments (Fig. 5B), expression was generally consistent with downregulation with *Glra1* showing moderately decreased expression in P7-injured animals and *Glrb* appeared to moderately downregulated in both P7- and P28-injured animals, although no change was statistically different from controls for any gene at either age.

#### Neuromodulatory neurotransmitters

The expression of serotonergic receptor genes *Htr1a* (5-HT_1a_), *Htr2a* (5-HT_2a_) and *Htr7* (5-HT_7_) was quantitated and the results are shown in Fig. 5A & B (black bars). In the L1–2 segments (Fig. 5A), serotonin receptor expression in P7-injured animals was generally decreased relative to controls where *Htr2a* (non-significantly) and *Htr7* (significantly) were both downregulated, but *Htr1a* showed a modest increase (not significant). In P28-injured animals, *Htr7* expression was significantly upregulated. In the L3–5 segment (Fig. 5B) the expression pattern was similar to the L1–2 segment. The expression of two serotonin receptors was moderately decreased (*Htr2a* and *Htr7*) and one was unchanged (*Htr1a*) in P7-injured animals, while in P28-injured animals only *Htr7* was moderately, though non-significantly, increased.

## Discussion

In this study we have used behavioural testing, retrograde axonal tracing and gene expression studies to begin to unravel the biological basis of our observation that at a particular stage of *Monodelphis* development (P28) spinal injured animals develop locomotion in the absence of supraspinal innervation. This is in contrast to animals injured at P7 who grow axons across the lesion and develop locomotor abilities that approach those of uninjured animals.

We have shown previously that opossums with complete spinal injury performed at four weeks of age (P28) are able to walk with weight-supporting hindlimb steps when adult. This was despite the complete absence of axon regrowth across the injury site and lack of restitution of supraspinal control, as evinced by the animals’ inability to swim using their hindlimbs. Part of the present study was an independent confirmation of previous observations [17], which was essential to repeat so that we could compare their locomotor abilities with the axonal tracer and gene expression studies.

The high degree of over-ground locomotor function described in *Monodelphi*s with spinal transections in a neonatal period is in contrast to the behaviour observed following a similar transection in the adult [17], [22] suggesting that P28-injured animals are somehow better able to access and utilize the spinal cord circuitry responsible for locomotion. This led to the speculation that in the absence of axonal regrowth across the site of injury local spinal circuitry may be modified or developed *de novo* as these animals grew into adulthood [17].

A notable finding of the present study is the extent of morphological remodelling of propriospinal neurons in the lumbar spinal cord, caudal to the injury site following a complete transection at T10, as shown by fluorescent labelling (Fig. 4). In P28-injured animals there were marked differences in the number of labelled neurons in the lumbar spinal cord, which have been quantified for the first time. In the L1–2 segments, an increase in the number of labelled propriospinal neurons was found, but in the L3–5 segments the number was decreased, suggesting a rearrangement of neuronal signalling architecture. In P7-injured animals, this remodelling was less pronounced: we found no change in labelling in the L1–2 segments, but a decrease, similar to P28 injured animals, in L3–5 segments.

The observed remodelling, especially in P28-injured opossums, where regrowth of fibres across the injury site does not occur, may be particularly important in mediating their behavioural responses following injury. The morphological changes of propriospinal circuitry detected by labelling experiments were strengthened by the expression studies of genes coding for different neurotransmitter receptors (see below).

### Locomotion and Propriospinal Labelling

In control adult *Monodelphis* the step durations for all limbs are approximately equal. This was not the case for P28-injured animals where unequal numbers of forelimb and hindlimb steps were observed (Fig. 1B). This suggests that in these animals the forelimbs and hindlimbs are operating at different cycle speeds and we speculate that their low but measurable regularity index represents the fact that four individual footsteps involving each limb may, by chance, fall in a coordinated manner, giving the impression of occasional coordination (Fig. 1C). This unequal usage of the limbs was a result not of a change in rate of hindlimb movements, but was due to shortening of forelimb step duration (Fig. 1D), suggesting that the forelimbs were required to compensate for the poor control and balance of the hindquarters. These timing irregularities also help to explain the variable level of observed coordination that led to a BBB score of 12.3±0.2 for P28-injured opossums (Fig. 1A).

In P28 injured animals injection of fluorescent tracer into the lumbar spinal cord did not result in any labelling of neurons in the brainstem or in the spinal cord rostral to the injury site, confirming the observations of our previous study [17] that regrowth of supraspinal or local fibres across the injury site does not occur in these animals and therefore can not account for the behavioural responses seen here. This lack of supraspinal drive is the major contributing factor to these animals’ inability to swim using their hindlimbs.

We have compared P28-injured animals with opossums injured earlier in development (P7). In spite of their substantially normal locomotion P7-injured opossums retained several deficits in their locomotor organisation. In the open-field testing all animals displayed abnormalities in foot placements leading to a lower than normal BBB score (Fig. 1A). On the treadmill, although full coordination was observed, differences in the timings of foot placements compared to controls led to differences in diagonal phase lags (Fig. 2C) suggesting that a reorganisation of step timing and execution had occurred.

Neuronal labelling was used to show that supraspinal and propriospinal neurons had grown across the injury site and into the L1–2 segments in the P7-injured animals. The mere presence of labelled supraspinal neurons does not itself necessarily indicate that they are functionally involved in any behavioural responses but the fact that animals injured at P7 walked with normal FL–HL coordination and were able to utilize their hindlimbs during swimming suggests that supraspinal control was likely to have been reinstated and was playing an important role in these animals.

**Figure 6:**
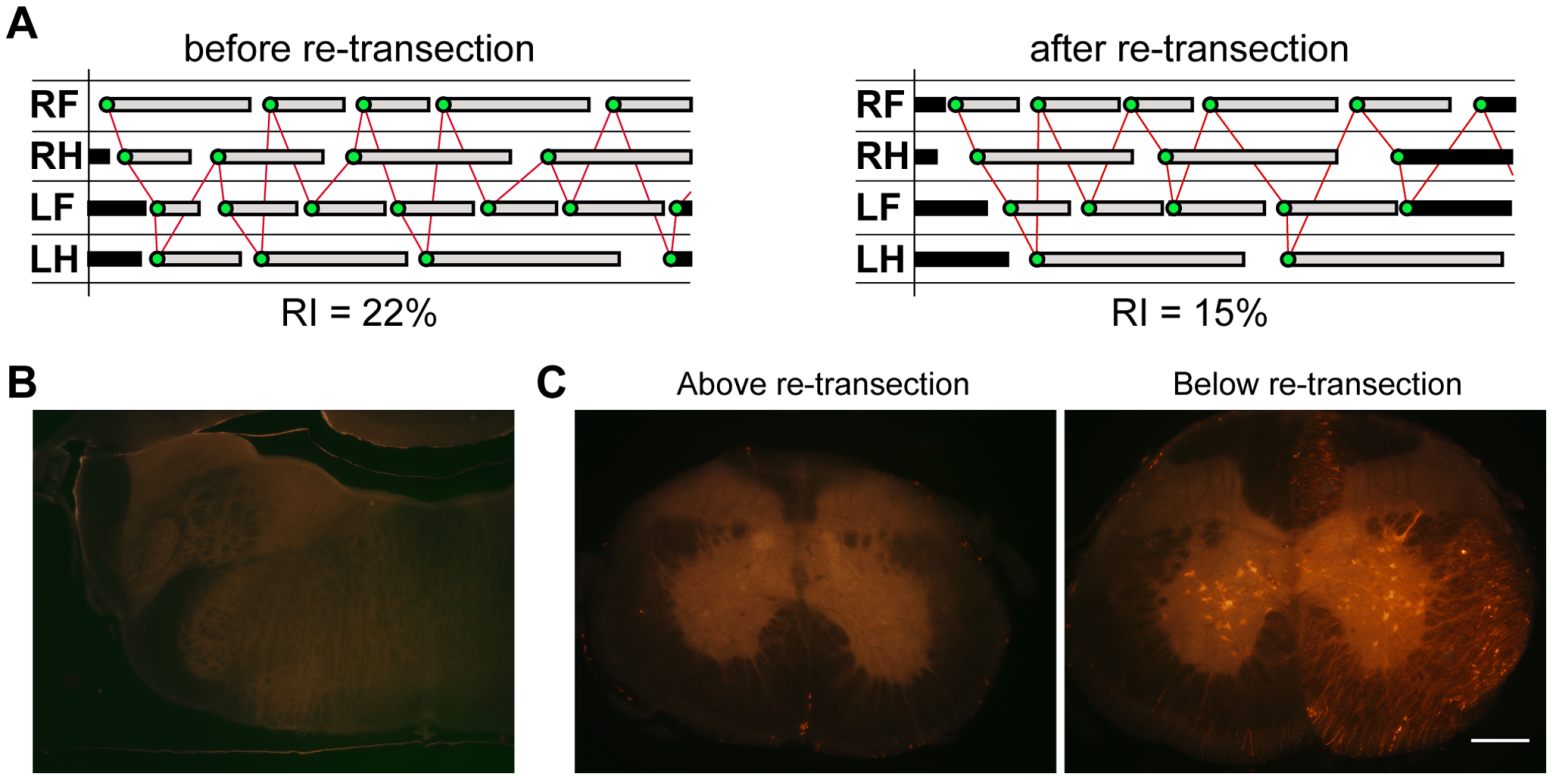
Re-transection of P28-injured opossum spinal cord. In adulthood the spinal cord of one P28-injured *Monodelphis* was re-transected through the centre of the injury site and allowed to recover for 3 weeks before behavioural and labelling studies were performed. ***A*** *:* Representative gait trace from P28-injured opossum in adulthood before (left panel) and after (right panel) re-transection. ***B*** *:* Image of section through the medulla of re-transected P28-injured animal following retrograde tracing. ***C*** *:* Images of sections through the spinal cord above (left panel) and below (right panel) the level of the re-transection. Re-transection was performed at the same level as the initial transection during the neonatal period and did not result in a second distinct transection site (data not shown).

To demonstrate further that the lumbar spinal cord, even when not connected to the brainstem, is capable of producing weight-bearing locomotion of the animal, we have performed a preliminary re-transection experiment on one P28-injured opossum. Results are illustrated in Fig. 6 and show that re-transection had little impact on the animal’s gait. Although a reduction in regularity index occurred, the animal was still able to perform weight-bearing stepping (Fig. 6A and Video S1). Spinal labelling after re-transection revealed that no fibres crossed the injury site (Fig. 6B–C). Taken together these observations suggest that the initial recovery was not due to spared axons, since surgically severing these would be expected to result in substantial loss of motor function [30].

### The Biological Significance of Gene Expression Studies

Gene expression studies following spinal cord injury are typically aimed at analysing changes in early response inflammatory or regeneration-associated genes at the centre of the injury site (e.g., [40], [41]). Changes in gene expression of neurotransmitter receptors are less commonly performed, and as a consequence only limited comparative information is available. However, two relevant papers are those of Wienecke *et al.,* [42] who studied gene expression changes in motor neurons following spinal transection and Aimone *et al.,* [43] who studied gene expression changes following spinal cord contusion; both studies were in adult rats. These are particularly important for comparison with our study because we were unable to carry out extensive spinal transection experiments in adult *Monodelphis* because of autophagy of the animals’ hindlimbs, which led to early termination of the experiments for ethical reasons [17].

Expression of several excitatory glutamate receptors subtypes changed following spinal injury, mostly in P28-injured animals where NMDA-1 was downregulated in the L1–2 segment and AMPA-2 and Glu-R metabotropic receptor-5 were upregulated in the L3–5 segment. NMDA-1 was found upregulated in the adult rat [42]. In P7-injured animals, two out of the three excitatory receptors appeared to increase their expression (NMDA-1 and AMPA-2) in the L1–2 segment but were not changed in the L3–5 segment. In the adult rat NMDA-1 was shown to increase its expression [40].

Glutamatergic transmission has been implicated as a key player in locomotor circuit activation in the cat [44] and rat [45] and it is thought that the primary effects are mediated through the NMDA receptors [44], [45]. These ionotropic glutamatergic receptors are also involved in fast network interaction between interneurons and motoneurons [46]. That NMDA agonism is commonly used as a circuit activator in studies into defining the essential components of the central patter generator [47]–[49] points to its importance as a positive modulator of locomotor output. Following injury, when other descending systems are lost, glutamatergic systems have been shown to be involved in the recovery of treadmill stepping [50] and preliminary studies by the same group demonstrated that receptor density was maintained in an upregulated state following injury [51], which was supported by Wienecke *et al.,* [42] who found upregulation of kainite and NMDA receptor subunits and associated proteins in lumbar motoneurons following adult spinal cord injury. This is similar to the finding in the present study where an upregulation at L1–2 of excitatory receptor genes in P7-injured spinal cord but contrasted with downregulation in P28-injured cord. The difference between P28-injured animals and those injured when adult may indicate important differences in circuit reorganisation at the two ages following spinal cord injury. For reasons mentioned above, it was not possible to do spinal injury experiments in adult *Monodelphis* and keep the animals for long enough for possible modifications to spinal circuitry to develop. A further contrast suggesting an increase in *Gria2* and *Grm5* in the L3–5 segments of P28-injured animals suggests that significant reorganization occurs not only between ages but also along the rostrocaudal axis of the cord. *Gria2* has been shown to be expressed more highly in interneurons than motoneurons in the lumbar cord [52], pointing to a potential difference in the balance of these neuronal subtypes in the lower lumbar segments of P28-injured animals.

The two major inhibitory neurotransmitters in the spinal cord are GABA and glycine. There was a general trend towards increased expression of one GABA- and two glycine-receptor subunits in P7-injured animals in the L1–2 segments which contrasted with the general decrease of these genes in P28-injured opossums. Upregulation of *Glra1* has been shown following injury to the adult rat [40]. These inhibitory receptors are thought to be expressed on interneurons in the lumbar cord [53] and consequently differences in their expression may suggest a reorganization of the regulation of central pattern generation.

The level of inhibitory drive may be modified after spinal injury [54]–[56]. An increase in GABA synthesis following spinal injury has been shown in cats [54] and an increase in GABA-A receptor subunits in certain motoneurons following injury in the rat has been observed [55]. Administration of GABA receptor blockers [57] or glycine receptor blockers [58] resulted in improved stance and locomotion suggesting that the inhibitory GABA- or glycinergic systems in the spinal cord interfere with locomotor generation. Similar increases in the amount of glycine receptor have been shown in the lumbar spinal cords of rats following spinal injury [56], similar to the P7-injured opossums. Locomotor training, which has positive benefits on the stepping ability of completely transected animals, normalizes the level of these inhibitory neurotransmitter receptors and synthesis molecules [55], [56], [59], pointing to a use-dependency of these systems.

The P28-injured animals in the present study were able to walk extremely well with no training of any kind. Taken alongside the evidence of decreased inhibitory transmitter receptor expression in their upper lumbar spinal cord this suggests that this downregulation may have helped to reduce the inhibitory drive, so that locomotion was possible. P7-injured animals generally displayed increased levels of inhibitory neurotransmitter receptor gene expression. It is possible that the presence of their re-established supraspinal innervation enabled them to overcome any increased inhibitory drive resulting from this overexpression. Overall the results suggest that there was a change in the balance between excitatory and inhibitory inputs from local spinal and supraspinal innervation in P7-injured animals compared to P28-injured animals.

Serotonergic receptor expression also changed following spinal cord injury. In the L1–2 segments of P7-injured animals, there was a general decrease in expression of *Htr2a* and *Htr7* but not *Htr1a*, suggesting a decrease in sensitivity to serotonin in these animals. This contrasted with the increased *Htr7* expression in P28-injured animals at L1–2 suggesting an increase in sensitivity.

Serotonergic systems in particular have been shown to make important contributions to descending pathways that mediate signals from the brainstem to all levels of the spinal cord [46]. The regulation of the neuromodulatory receptors in the injured spinal cords here may be a reflection of the differing supraspinal drive that is present in these animals. It is likely that at least a portion of the brainstem neurons whose axons span the lesion site in the P7-injured animals would be serotonergic since retrograde axonal labelling consistently labels neurons in the areas where these systems are known to originate, the raphe nuclei and sub coeruleus respectively [60], [61].

However, since only a proportion of these regrow following injury at P7, the decrease in the neuromodulatory receptor gene expression could be an adaptation reflecting this limited level of supraspinal input. In the P28-injured opossums, in which no supraspinal drive was present, there was a decrease in expression of this serotonin receptor, and no change in any of the others that were examined. This change may reflect downregulation resulting from the lack of use of this system.

Previous studies have also revealed that following spinal injury adaptive changes occur for many receptors in the lumbar spinal cord. Giroux *et al.,* [62] found that in the first weeks after injury there was an upregulation in receptors of the adrenergic and serotonergic systems, as measured by autoradiographic receptor binding studies, but these later declined to control levels. The authors speculated that this change in receptor regulation demonstrated the continued importance of these systems after complete spinal transection [62]. Increased expression of a wide array of neuromodulatory receptors were found in the late phase after SCI in adult rat, analysed by PCR [63].

### The Role of Propriospinal Input

More research is becoming focused on the role that the isolated spinal cord itself may play in recovery. Recent studies have indicated that propriospinal remodelling plays an important role in recovery of function in adult animals with incomplete spinal lesions [64], [65]. Propriospinal relay connections have been demonstrated to reconnect pyramidal cells in the cortex with their target lumbar motoneurons [64] and mediate the reinstatement of supraspinal motor control despite the absence of any direct supraspinal innervation [65]. However, studies such as these rely on animals with partial lesions where spared tissue provides a bridge through which reconnections are made. Adult animals with complete spinal transections do not recover to the same degree unless external stimulation is given in the form of cutaneous feedback [56], [66], [67], serotonergic agonist administration [68], electrical stimulation [69], or a combination of each [70]–[72].

In contrast to other reports in which weight supported stepping has been reinstated following injury, the recovery of spinal injured opossums in the present study is independent of any interventions and consistently occurs in overground locomotion as well as on the treadmill.

### Limitations of the Study

#### Spinal injury in adult animals

One limitation to the approach taken in the present study is the inability to perform complete spinal transections in control, adult opossums and keep the animals long enough to study their locomotion post-injury. This experimental approach was attempted but unfortunately the animals that were injured for the very first time while adult were found to chew at their insensate hindlimbs and tail so aggressively that studies had to be halted a week post injury (also described in [17]). The autophagia continued in spite of attempts to dissuade the animals by painting their hindlimbs with various foul-tasting liquids normally used to stop people biting their nails. This group of animals would have added significant value to the comparison with P7-, P28-injured opossums and those that were re-transected as adults, particularly in respect to the labelling and gene expression studies. However we were able to use this difficulty for comparative purposes in observations of re-transected animals. Thus the P28-injured animal, when re-transected as adult, did not chew on its hind limbs indicating differences in its spinal sensory circuits. Instead results of similar experiments in *Didelphis virginiana* and/or rats have been used in the present study for comparison where appropriate.

#### Labelling protocol

In a previous study [17] where the sole intention of the spinal labelling experiments was to establish whether or not re-growing supraspinal axons (i.e., originating in the brainstem neurons) could be found, bilateral injections of fluororuby were used and the entire brainstem was sectioned and counted to establish the extent of the regrowth. In the current study, however, unilateral injections were employed so that propriospinal neurons, both ipsilateral and contralateral that projected into the injection area could be counted and mapped to assess remodelling rather than regrowth. As all animals were injected in a similar manner, a direct comparison of results obtained for labelled neurons between different groups should reflect differences in their connectivity.

#### qRT-PCR gene expression

The *Monodelphis* genome is not well annotated and no commercially available molecular tools are available. An initial study was performed using a commercially available Profiler Human Neurotransmitter Receptor PCR array plate (Qiagen) to screen for differentially expressed genes. However there was a lack of consistent and repeatable hybridization with *Monodelphis* cDNA, leading to large variability in control values so that no statistical differences between operated and control animals could be detected. Other studies using more conventional laboratory species have successfully employed genetic screens (usually in the form of microarrays [40]–[43], [52]). We limited our studies to three representative genes from each category of transmitters: excitatory, inhibitory and neuromodulatory. It is possible that other neurotransmitter receptors might have shown other differences. An immunocytochemical demonstration of some of these gene products would have added important information. Unfortunately none of the commercially available antibodies to these antigens from other animal species cross-react with *Monodelphis* tissue.

### Conclusions

The experiments described in the present study provide the beginnings of a basis for understanding the mechanisms that allow developing quadrupedal animals to recover from a complete spinal transection and be able to exhibit weight-bearing locomotion in the absence of any axon growth across the lesion site and indicate the important role that propriospinal remodelling may play [30]. We are primarily interested in the biological mechanisms underlying the substantially normal development that occurs following spinal cord injury when the lesion is made very early in development. We do not make any specific claims about how this approach to spinal cord injury might contribute to new therapies in spinal patients, although this may be possible once the mechanisms are better understood. From the present study it seems likely that the observed weight bearing and alternating hindlimb movements in spinally transected animals are primarily a function of the sensory input from the hind limbs during over ground walking, as these movements are lost when the animal is placed in water. However, the forward locomotion may be generated by the stretching of trunk and limb muscles and joints when the animal initiates movement with the forelimbs. This raises concerns about the suitability of rodents and other quadrupeds for studies of spinal cord injuries, where an attempt may be made to apply the results to human patients.

The present study suggests that significant weight bearing and forelimb–hindlimb movements can occur even in the absence of neural connections across a lesion and emphasizes the need for retrograde labelling to show whether or not apparent improvements in the BBB or BMS scores do show evidence for supraspinal re-innervation following spinal cord injury (e.g., [33], [73]–[77]). If weight-bearing locomotion in spinally injured animals is indeed initiated by the forelimbs then this will be of limited value for patients. A bipedal model of spinal cord injury would be more appropriate; this is approached by the use of robotic harnesses in rats, which oblige the rats to walk on their hindlimbs [15], [78], otherwise species that are normally bipedal need to be considered.

## Supporting Information

Figure S1 **Labelling of brainstem neurons.** ***A***: Schematic of labelling procedure. ***B***: Number of labelled brainstem neurons in control, P7-injured and P28-injured opossums. Mean ± sem; *P≤0.05 vs control; ^#^ *P*≤0.05 vs P7-inj, by One-way ANOVA.
(TIF)

Video S1 **Re-transection of P28-injured opossum.** Video footage P28-injured opossum before and after re-transection of the spinal cord at the same site as the original transection. A control opossum walking on the treadmill at the same speed is shown for comparison in order to highlight the decreased FL–HL coordination.
(MP4)
