# Supplementary figures and images for: Weight-Bearing Locomotion in the Developing Opossum, Monodelphis domestica following Spinal Transection: Remodeling of Neuronal Circuits Caudal to Lesion
Source: PLoS One. 2013 Aug 12;8(8):e71181. doi: 10.1371/journal.pone.0071181 (PMC3741377; doi:10.1371/journal.pone.0071181)

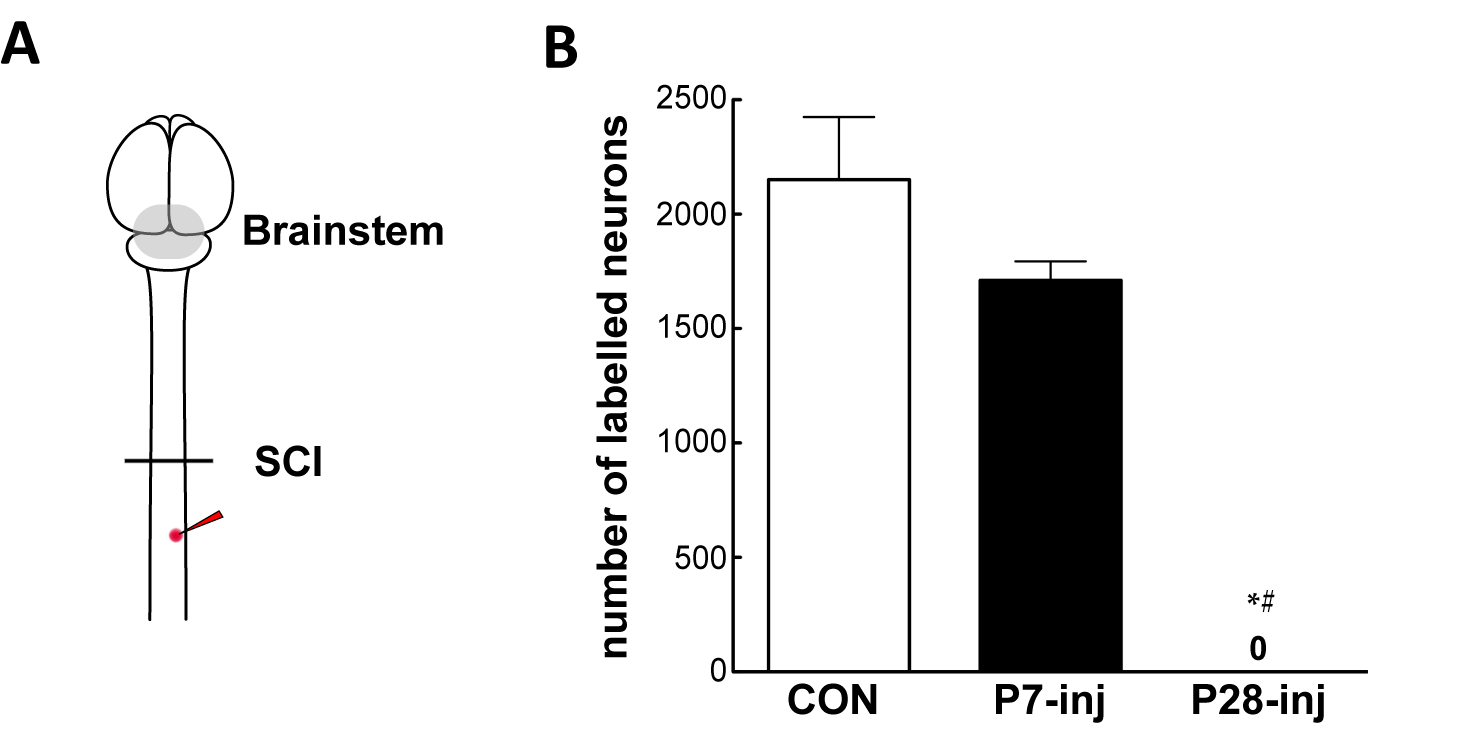

Supplement: Figure S1 — Labelling of brainstem neurons. A: Schematic of labelling procedure. B: Number of labelled brainstem neurons in control, P7-injured and P28-injured opossums. Mean ± sem; *P≤0.05 vs control; # P≤0.05 vs P7-inj, by One-way ANOVA. (TIF) [file pone.0071181.s001.tif]
